# Multi-UAV Path Planning in GPS and Communication Denial Environment

**DOI:** 10.3390/s23062997

**Published:** 2023-03-10

**Authors:** Yahao Xu, Yiran Wei, Di Wang, Keyang Jiang, Hongbin Deng

**Affiliations:** School of Mechatronical Engineering, Beijing Institute of Technology, 5th South Zhongguancun Street, Beijing 100081, China

**Keywords:** multi-UAV cooperative, reinforcement learning, path planning, visual perception, GPS denial

## Abstract

This paper proposes a feature fusion algorithm for solving the path planning problem of multiple unmanned aerial vehicles (UAVs) using GPS and communication denial conditions. Due to the blockage of GPS and communication, UAVs cannot obtain the precise position of a target, which leads to the failure of path planning algorithms. This paper proposes a feature fusion proximal policy optimization (FF-PPO) algorithm based on deep reinforcement learning (DRL); the algorithm can fuse image recognition information with the original image, realizing the multi-UAV path planning algorithm without an accurate target location. In addition, the FF-PPO algorithm adopts an independent policy for multi-UAV communication denial environments, which enables the distributed control of UAVs such that multi-UAVs can realize the cooperative path planning task without communication. The success rate of our proposed algorithm can reach more than 90% in the multi-UAV cooperative path planning task. Finally, the feasibility of the algorithm is verified by simulations and hardware.

## 1. Introduction

A UAV has the following advantages: concealment, mobility, and low cost. As the application scenarios become more and more extensive, the UAVs’ flight environment becomes more complex. Currently, improving the autonomous path planning capability of UAVs has become a critical element in the UAV’s mission decision-making system, which plays a crucial role in ensuring flight safety and improving the efficiency of UAVs.

With the wide application of UAVs in tracking disaster rescue and environmental exploration, UAV path planning algorithms have become increasingly important [1,2]. However, complex and diverse real-world flight environments present significant challenges to the robustness of multi-UAV systems. Errors in path planning can cause considerable damage to people or other property.

Traditional UAV path planning methods include the artificial potential field method [3,4], A* algorithm [5], genetic algorithm [6], and MPC [7]. These methods are divided into three steps: environment mapping, path planning, and motion control. It takes much time to build environment mapping, so traditional path planning methods are often unsuitable for real-time UAV navigation tasks.

The traditional planning algorithm has the following defects: complex operations, low real-time performance, can easily cause a local optimum, and the inability to quickly respond to complex environmental changes and incorporate flexible learning. In the face of the problems existing in the traditional path planning algorithm, reinforcement learning is applied to path planning as an artificial intelligence algorithm. This learning method converts the sequential decision problem into a Markov model. The core of the algorithm is to establish the mapping between the environment state and the state–action value function via the interaction between the agent and the environment and then to obtain the optimal state–action value function. Finally, the best action sequence is obtained. Observing that a UAV’s reactive navigation can be viewed as a sequential decision problem, more and more researchers are considering using dynamic programming properties for path planning based on reinforcement learning methods. The reinforcement learning method does not need to build a map in advance, and the UAV can dynamically avoid obstacles and plan the path in real time.

**Figure 1:**
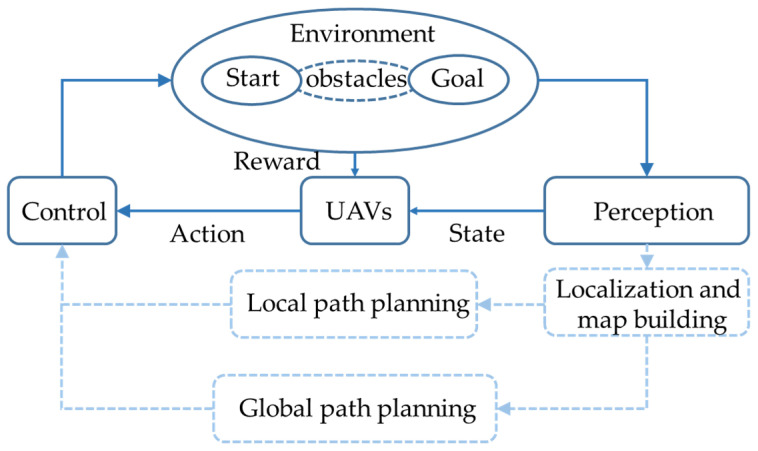
DRL-based navigation system. The solid line part of the picture is the method using reinforcement learning as path planning, and the dotted line is the traditional path planning method.

Figure 1 shows that the path planning method using reinforcement learning differs from the traditional method. The traditional method requires perception localization and mapping, and then the navigation of the UAV is carried out after the path is planned. However, reinforcement learning directly performs actions according to the policy, connecting the motion trajectories of the UAV to obtain the path.

Xie et al. [8] proposed a reinforcement learning algorithm based on a heuristic function and experience replay mechanism with a maximum average reward value. The algorithm has good learning efficiency, convergence speed, and significantly improved training performance. Cui, Z.Y., et al. [9] proposed a multi-layer path planning algorithm based on reinforcement learning techniques. Compared with classical Q-learning, the proposed multi-layer algorithm has obvious advantages, which can collect global and local information.

The multi-UAV cooperative has a significant development trend in future air combat. Compared with a single UAV, a multi-UAV has higher combat effectiveness and stronger combat ability. In the process of multi-UAV cooperative flight, path planning technology can provide path guidance for UAVs, which is one of the key technologies for realizing the UAVs’ cooperative operation. Cooperative path planning can obtain the optimal path satisfying the UAV performance constraints and time cooperation constraints, which is an essential guarantee for the autonomous flight of multiple UAVs. A reasonable path can save the cost of UAV operation and increase the success rate of completing the reconnaissance mission.

For multi-UAV path planning, some researchers [10,11] have proposed solutions for multi-UAV collision avoidance systems based on centralized algorithms. Centralized algorithms rely on a central server, which is used to communicate with each agent and generate global control commands based on the observations of all UAVs. Later, the MADDPG [12] algorithm was proposed to solve the problem of agent heterogeneity. MADDPG uses observations from multiple agents as critics and observations from each agent as actors.

Maintaining stable communication in large-scale complex scenarios is often difficult or impossible due to radio interference and masking areas. The dependence of centralized multi-UAV systems on communication makes them challenging to deploy in practice. Although the MADDPG method uses each agent’s observations in the execution phase, it still needs the agents to share the observations in the train phase. Therefore, only distributed control with independent policies can be used for communication denial environments.

Meanwhile, all the above literature assumes that the UAV knows the precise location of the target, and the reward function is set according to the distance between the UAV and the target. However, in a GPS-denied environment, the UAVs cannot obtain the position of the target, which means that the previous path planning method is invalid.

The causes of UAV communication and GPS denial vary [13,14] and include GPS spoofing attacks, data interception attacks, denial of service attack malware, infection attacks, and man-in-the-middle attacks.

Aiming at the path planning problem of having an unknown target location in a GPS-denied environment, this paper designs a path planning algorithm with a visual navigation function for UAVs. Firstly, in the GPS-denied environment, the accurate location of the target cannot be obtained, and the traditional path planning algorithm fails. In this paper, the UAV uses the YOLOv5 [15] algorithm for target detection to determine the target position. Then, feature and image fusion are used as the input of the reinforcement learning algorithm for path planning. Second, a distributed control approach based on independent policies is used, where each UAV is an independent agent making its own decisions based on the obtained observations. Finally, simulation and hardware-in-the-loop experiments are carried out. The results show that our proposed method is comparable to the success rate of the known precise target location. The main contributions of this paper are as follows:

- To solve the problem of UAV path planning in GPS blocked environment, we introduce target recognition algorithms based on the reinforcement learning method to realize multi-UAV path planning. Compared with the direct use of the end-to-end reinforcement learning method, the results obtained from image recognition are fused with the original image as an observation. The reinforcement learning algorithm can make the UAV perform the corresponding action according to the observation and finally connect the trajectory of the UAV, which can realize the path planning of multiple UAVs without precise target locations.
- Considering the problem of path planning for multiple UAVs in the blocked environment, we proposed a distributed control method based on an independent policy. This method does not require the UAVs to communicate with each other. Each UAV uses its own observation results to make flight decisions.
- A simulation platform is built, and hardware-in-the-loop experiments are carried out on the hardware to verify the feasibility of the algorithm. Experimental results show that the proposed algorithm can realize the cooperative path planning of multiple UAVs without precise target locations, and its success rate is close to that of the known precise target location.

The rest of the paper is organized as follows: Section 2 reviews related works. Our approach is described in Section 3. Section 4 describes the experimental environment and analyzes the experimental results. Finally, the conclusion is summarized in Section 5.

## 2. Related Works

**Figure 2:**
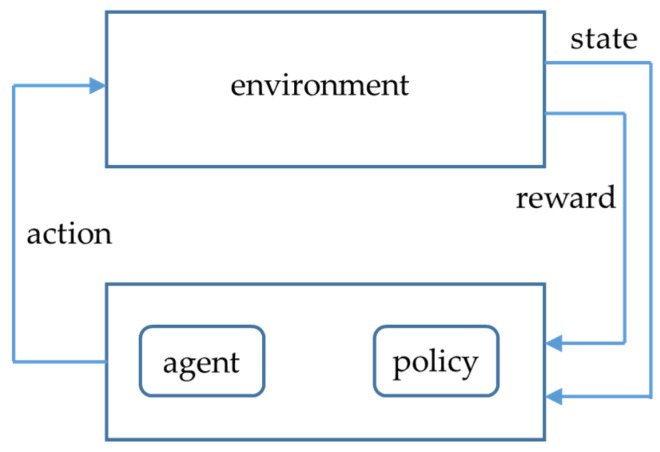
The concept of reinforcement learning.

Researchers in recent years have paid more and more attention to the path planning algorithm based on reinforcement learning [16]. Reinforcement learning is a process of mapping states (State) to actions (Action) and maximizing rewards (Reward) in the current environment. In this setting, the agent obtains an observation of the environment and can operate via a defined set of actions. After each executed action, the agent will receive the corresponding reward, and the goal is to find the optimal policy. The learning process of reinforcement learning is given in Figure 2.

Path planning can be regarded as a sequential decision problem, which is the same idea as the Markov decision process (MDP). MDP is the key to the reinforcement learning algorithm, which is characterized by a quadruplet consisting of state space S, action space A, transfer probability distribution P, and reward R, where policy π is a mapping of the actions, a, that will be selected in any state. The discounted cumulative reward corresponding to this sample at a given policy is given by Equation (1):

(1) $$G_{t}=R_{t+1}+\gamma R_{t+2}+\cdots =\sum_{k=0}^{\infty }\gamma^{k}R_{t+k+1}$$

where γ is the discount rate, which is used to measure the importance of immediate and subsequent rewards.

Accordingly, the value function starting from state s and following policy π denoted by $V^{\pi }\left(s\right)$ is given by

(2) $$V^{\pi }\left(s\right)=E_{\pi }\left[\sum_{t=0}^{\infty }\gamma^{t}r\left(s_{t},a_{t},s_{t+1}\right)|a_{t}∼\pi \left(\cdot |s_{t}\right),s_{0}=s\right]$$

and given action a, the Q-value is defined as follows.

(3) $$Q^{\pi }\left(s,a\right)=E_{\pi }\left[\sum_{t=0}^{\infty }\gamma^{t}r\left(s_{t},a_{t},s_{t+1}\right)|a_{t}∼\pi \left(\cdot |s_{t}\right),s_{0}=s,a_{0}=a\right]$$

Given a known state transition probability distribution $p\left(s_{t+1}|s_{t},a_{t}\right)$ and the reward $r\left(s_{t},a_{t}\right)$, the following is obtained from the Bellman equation:

(4) $$V^{\pi }\left(s_{t}\right)=\sum \pi \left(a|s_{t}\right)\sum p\left(s^{\prime }|s_{t},a\right)\left[r\left(s_{t},a\right)+\gamma V^{\pi }\left(s^{\prime }\right)\right]$$

The agent explores the environment with the aim of obtaining the optimal state–action value function $Q^{*}\left(s,a\right)$ and tends to obtain the greatest reward when it chooses the action that yields the optimal state–action value function. The optimal state–action value can be obtained by the following.

(5) $$Q^{*}\left(s_{t},a_{t}\right)=\sum_{s^{\prime }}p\left(s^{\prime }|s_{t},a_{t}\right)\left[r\left(s_{t},a_{t}\right)+\gamma \max Q^{*}\left(s^{\prime },a^{\prime }\right)\right]$$

When the optimal Q is obtained, the optimal policy can be generated. The reinforcement learning method explores and learns via the “try-failure-try” cycle method and finally achieves the avoidance of obstacles and path planning. Reinforcement learning technology has been well developed in path planning. This method only needs to train environmental samples to obtain a path planning model. In 2005, Michels J et al. [17] applied the reinforcement learning method to the path planning system and trained the appropriate path planning policy via the model. Xie et al. [18] used the double DQN algorithm to achieve obstacle avoidance planning in an indoor environment. Vamvoudakis et al. [19] also adopted the reinforcement learning method in the field of obstacle avoidance for agents. Still, the results show that the feature quality of the selected samples greatly impacted the effect of obstacle avoidance. Kulkarni et al. [20] used the goal-driven intrinsic motivation deep reinforcement learning method to learn the guiding behavior in a real-time environment. They improved the convergence speed of the obstacle avoidance algorithm in complex environments.

In addition, the path planning problem for UAVs in GPS-weak or GPS-denied environments has also drawn more and more attention from researchers. Without a locating system, UAVs must rely on information collected by RGB cameras, infrared cameras, radar, laser, and other sensors to plan a path. To solve these problems and achieve navigation, Zhu et al. [21] first applied deep reinforcement learning (DRL) to visual navigation. They proposed a set of visual navigation architecture. In this framework, the UAV can autonomously navigate to the target position only by using the input image’s information, which has a significant role in promoting the field of visual navigation. However, the limitation of this framework is that it can only be applied to the case of a single UAV. According to the work of Zhu et al., Chen et al. [22] added an LSTM before the policy generation layer to save the previous path, but the algorithm is unstable. Siriwardhana et al. [23] used a VUSFA (variational universal successor features approximator) to solve the problem of visual navigation in complex environments. This algorithm has the advantage of being adaptable to tasks other than navigation. Siriwardhana et al. [24] proposed a method named HAUSR (hybrid asynchronous universal successor representations). The authors combined it with the asynchronous advantage actor-critic algorithm (A3C) to improve the adaptability of the model relative to new scenarios. However, the performance of the proposed method degrades significantly during long navigation tasks. Qie et al. [25] proposed a reinforcement learning method based on a MADDPG algorithm: synchronous goal assignment and path planning (STAPP). The algorithm is a multi-agent reinforcement learning algorithm. The MADDPG framework trains the system to simultaneously solve goal assignment and path planning according to the corresponding reward structure. The summary is shown in Table 1.

Based on the above analysis, common path planning algorithms assume the precise position of the target and design the reward function according to the target’s position. However, this is not accessible in GPS-denied environments, which means that many path planning algorithms cannot be used. The current UAV path planning algorithm has considered the problem of GPS denial. However, it is limited to path planning for a single UAV. This means that the problem of UAV cooperation in the communication denial environment is not considered. It is necessary to propose a multi-UAV path planning algorithm in an environment without GPS and communication.

## 3. Proposed Method

### 3.1. Problem Definition

**Figure 3:**
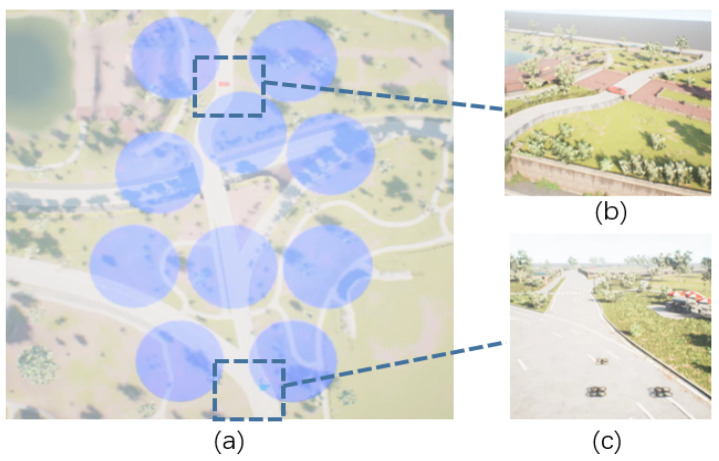
Scenario of multi-UAV path planning. (**a**) is the top view, (**b**) is the target position, and (**c**) is the initial position of the multi-UAV. The blue area is the defensive area, which is only used as a schematic for the reader, and it is not visible in the actual training and flight.

Our assumption is in the military domain, where UAVs need to traverse enemy positions and reconnaissance. The enemy often has air defense radars and other equipment, and we want the UAVs to avoid these air defense radars and fly to the destination. The detection area of this kind of air defense radar is generally conical and cannot be viewed visually. We assume in training that the UAVs know the position of the air defense radar, but the target may shift, so we can only obtain an ambiguous position of the target. The UAVs can only obtain environmental information in an unknown environment via first-person images, as shown in Figure 3:

1. The UAV should avoid the defense zone during the flight. If the UAVs enter the defense zone, the UAVs will be destroyed or captured.
2. The defense zone is generated by electromagnetism and cannot be observed visually.
3. Due to GPS denial, the UAV can only know the area of the target but cannot know the precise location of the target.
4. Due to the denial of communication, UAVs cannot share information and can only perceive the position and status of other UAVs via the onboard camera.

### 3.2. Feature Fusion PPO

**Figure 4:**
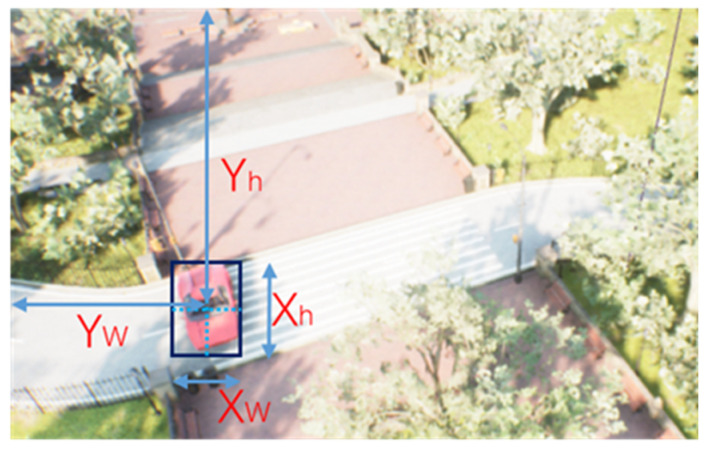
Location and size information of the recognition box obtained by the YOLOv5 algorithm. $X_{h},X_{w}$ represent the size of the object in the image, and $Y_{w},Y_{h}$ represent the position of the object center in the image. They are normalized values, which are obtained by taking the number of target pixels divided by the height or width of the image.

Standard end-to-end path planning algorithms use the images obtained by the onboard camera as input and design the reward function by utilizing the distance between the UAV and the target. However, the reward function containing the location cannot be used in the GPS-denied environment, because there is no precise value with respect to the target’s location. The end-to-end path planning algorithm fails. To solve the above problems, we introduce the object detection algorithm, which uses the area and position of the image recognition box to determine whether the UAVs have reached the target. This solves the problem of path planning without the precise location of the target. When the area of the detection box of the target in the image is greater than a certain value, this means that the UAV has flown to the target position and completed the path planning operation, as shown in Figure 4.

Common object detection algorithms are mainly divided into two categories: one is the second-order detection method represented by the RCNN [26,27,28] series, and the other is the first-order detection algorithm represented by the YOLO [15,29] series.

**Figure 5:**
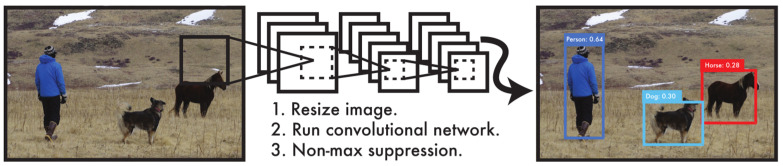
The framework of the YOLO algorithm [29].

YOLO is an object detection algorithm. Object detection aims to find all regions of interest in an image and determines the location and class probability of these regions. YOLO reformulates object detection as a regression problem. It applies a single convolutional neural network (CNN) to the entire image, splits it into grids, and predicts the class probabilities and bounding boxes for each grid. The YOLO algorithm has fast detection speed. Since the detection problem is a regression problem, there is no need for a complex pipeline. The principle of the YOLO algorithm is shown in Figure 5.

Currently, YOLOv5 has reached SOTA in terms of detection accuracy and speed. We chose YOLOv5 as the image recognition algorithm in multi-UAV path planning, and YOLOv5 adopts Focus and C3Net on the backbone network. YOLOv5 designs two different C3Nets for the Backbone and Detect head. The loss function of YOLOv5 is as follows.

(6) $$loss=\lambda_{1}L_{cls}+\lambda_{2}L_{obj}+\lambda_{3}L_{loc}$$

$L_{cls},L_{obj},L_{loc}$ denote category loss, positive and negative sample loss, and location loss, respectively. $\lambda_{1},\lambda_{2},\lambda_{3}$ denote the equilibrium coefficients. In this paper, we sampled the target position, labeled it, and then trained the YOLOv5 algorithm to obtain the detection weight.

**Figure 6:**
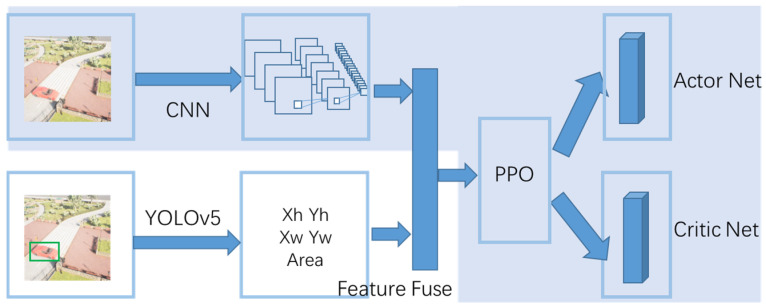
FF-PPO architecture. The input of the PPO algorithm contains two parts: One is the image acquired by the UAV, and the other part is the location information of the target acquired by the YOLOv5 algorithm. $X_{h},Y_{h},X_{w},Y_{w}$ represent the position of the object, and area denotes the area of the target identified by the YOLO algorithm in the image.

However, directly using the detection box will introduce a serious partial observable problem. At the beginning of the task, the UAV cannot find the target, resulting in a constant observation value of 0, which means that no valuable observation can be obtained. In this case, it is difficult for the UAV to explore and complete the path planning task. We use a fusion of images and features as input to our algorithm, named the FF-PPO algorithm. We provide the input of reinforcement learning to both the result of target recognition and the original image: using the original image to make the UAV perceive the air defense zone and other UAVs and using the result of target recognition to perceive the location of the target. These two are fused for a full connection, which increases the perception capability of the UAV and can alleviate partial observability. The two parts of the inputs are then sent to the PPO algorithm [30] to obtain the policy and state value functions. The architecture is shown in Figure 6. The shaded part is the common path planning algorithm that uses the PPO algorithm, but the limitation of this method is that the exact location of the target must be known.

The PPO algorithm is a variation of the policy gradient algorithm. The policy gradient algorithm is an on-policy algorithm; that is, the agent’s policy for exploring to collect data and the policy for learning are the same. The on-policy algorithm updates the policy’s parameters immediately after the agent interacts with the environment. It then uses the new policy to interact with the environment to collect new data. Therefore, the data collected in the same policy algorithm can only be used once, significantly reducing efficiency. Aiming at the problem of the low data utilization rate of the on-policy algorithm, an off-policy algorithm has been proposed. The off-policy algorithm is modified by the importance sampling method in order to reduce the error caused by different policies.

The policy gradient algorithm is susceptible to the step-size parameter, but choosing the step size is often difficult. In the policy optimization of reinforcement learning, we want the policy to gradually converge to the optimal policy with each update. If the number of steps per update is too small, which makes the convergence slow, and if the number of steps per update is too large, which makes the convergence process easily oscillate, the target function proposed by PPO can be iteratively updated with several samples in multi-round training. This solves the problem that the step size is difficult to determine and the update difference is too large in the policy gradient algorithm.

**Figure 7:**
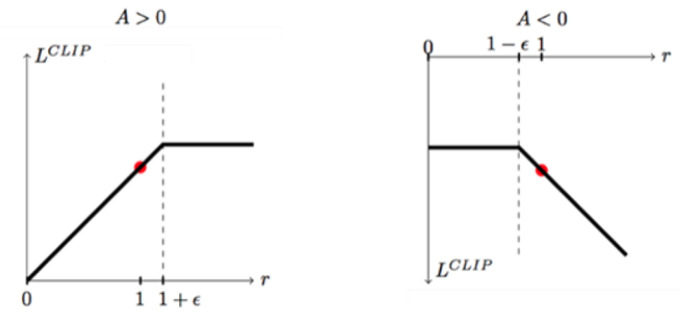
The clip function for the PPO algorithm. When the difference between the old policy and the new policy is less than 1 or slightly greater than 1, the clipping function does not work; only when the difference between the old policy and the new policy is too large will the clipping function limit the difference between the old policy and the new policy to 1+ε.

Figure 7 is the key to restricting policy updates, which is essentially a segmentation function. When the difference between the old and new policies is less than 1+ε, the update step of the old and new policies is not too large and is not processed. When the difference between the old and new policy steps is greater than 1+ε, the update of the old and new policy is forcibly restricted to 1+ε in order to avoid a difference that is too large in one update, which leads to the instability of the policy. When the dominance function is negative, the clipping function on the right is then used [30].

The loss function [30] for PPO is as follows:

(7) $$L^{CLIP}\left(\theta \right)=E_{t}\left[\min\left(r_{t}\left(\theta \right){\hat{A}}_{t},clip\left(r_{t}\left(\theta \right),1-\varepsilon ,1+\varepsilon \right){\hat{A}}_{t}\right)\right]$$

where θ is the policy parameter, and $E_{t}$ denotes the empirical expectation over timesteps. $r_{t}$ is the probability ratio under the new and old policies. ${\hat{A}}_{t}$ is the estimated advantage at time t. ε is a hyper-parameter, usually 0.1 or 0.2.

The PPO algorithm uses the actor–critic framework, and the loss function [30] of the critic is as follows:

(8) $$L\left(\phi \right)=-\sum_{t-1}^{T}\left(\sum_{t^{\prime }>t}\gamma^{t^{\prime }-t}r_{t^{\prime }}-V_{\phi }\left(s_{t}\right)\right)$$

where ϕ is the critic parameter, and V denotes the state function. The implementation process of the FF-PPO algorithm is shown in Algorithm 1.
**Algorithm 1.** Feature Fusion PPO Algorithm Initialize policy net θ
 Initialize critic net ϕ
 Initialize hyper-parameter (γ,ϕ,θ,M,N)
 **for**
$i\in \left\{1,\cdots ,N\right\}$
**do**
  Get the target position and area by YOLOv5  Then join with the image as a tuple $s_{t}$
  Run policy $\pi_{\theta }$ for T timesteps, collecting $\left\{s_{t},a_{t},r_{t}\right\}$
  Estimate advantages
${\hat{A}}_{t}=\sum_{t^{\prime }>t}\gamma^{t^{\prime }-t}r_{t^{\prime }}-V_{\phi }\left(s_{t}\right)$
  $\pi_{old}\leftarrow \pi_{\theta }$
  **for**
$j\in \left\{1,\cdots ,M\right\}$
**do**    $L^{CLIP}\left(\theta \right)=E_{t}\left[\min\left(r_{t}\left(\theta \right){\hat{A}}_{t},clip\left(r_{t}\left(\theta \right),1-\varepsilon ,1+\varepsilon \right){\hat{A}}_{t}\right)\right]$
    Update θ by a gradient method $L^{CLIP}\left(\theta \right)$
  **end for**  **for**
$j\in \left\{1,\cdots ,B\right\}$
**do**    $L\left(\phi \right)=-\sum_{t-1}^{T}\left(\sum_{t^{\prime }>t}\gamma^{t^{\prime }-t}r_{t^{\prime }}-V_{\phi }\left(s_{t}\right)\right)$
    Update ϕ by a gradient method  **end for** **end for**

### 3.3. Independent Policy PPO

A widely used paradigm with respect to multi-agent reinforcement learning is parameter sharing or the centralized evaluation of decentralized execution, which requires communication between the observations of agents.

**Figure 8:**
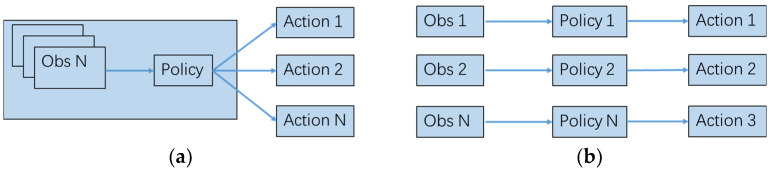
Centralized and independent strategies. (**a**) represents a centralized strategy, and (**b**) represents an independent strategy, which can realize distributed control without communication.

The independent policy paradigm treats all other agents and the environment as a whole. The action changes in other agents lead to the instability of the environment. This method requires a small number of agents and has higher requirements for each agent’s perception ability and decision-making ability. We use the independent policy PPO algorithm, where each UAV corresponds to its policy. The UAVs do not need to communicate with each other and only need to perform actions based on their observations, as shown in Figure 8.

## 4. Experiment

### 4.1. Environmental Model

AirSim [31] is a simulation platform developed by Microsoft for autonomous vehicles such as UAVs and self-driving cars. Third-party vendors or research users can use these tools to produce realistic landscapes.

We use AirSim to build an aerial scene, including the target and the defense zone. The UAVs need to search for a path to approach the target within a gap in the defense zone and avoid being destroyed when entering the defense zone. We assume that the defense zone is a fixed area and is visually unobservable.

We first use AirSim to obtain 10,000 target images and then use AirSim’s function to obtain the target locations as labels. We train the weights for target detection using the default hyperparameters provided by YOLOv5 [15] and save the obtained weights. This is the preparation step for performing FF-PPO. In path planning, the UAV acquires one observation, and we use the target detection algorithm to identify the acquired image, in which the weights of the YOLO algorithm are fixed and are not updated anymore.

### 4.2. Settings for Observations, Action Spaces, and Rewards

In this paper, the observation space of the UAV is set as a tuple of images and vectors. The image is the forward first-person visual information of the UAV, and the size is 84 × 84 × 3. The vector is the coordinate and area value of the recognition box obtained after the YOLOv5 algorithm recognition.

**Figure 9:**
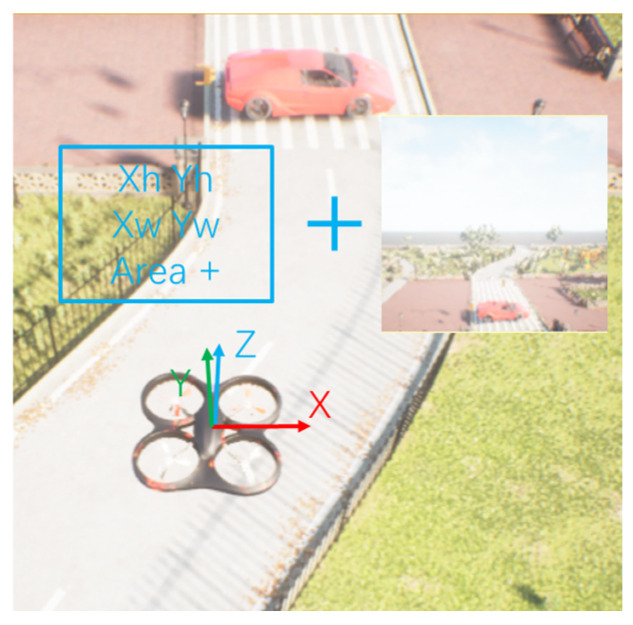
Observation and action space of the UAV. At each step, the UAV takes gray images and identifies information as observations and then performs actions accordingly.

The action space of the UAVs is continuous, which controls the movement direction of the three XYZ axes of the UAVs. The range is (−1, 1). According to the style of OpenAI-gym [32], the action space is Box (−1, 1, shape = (3,)), as shown in Figure 9.

**Figure 10:**
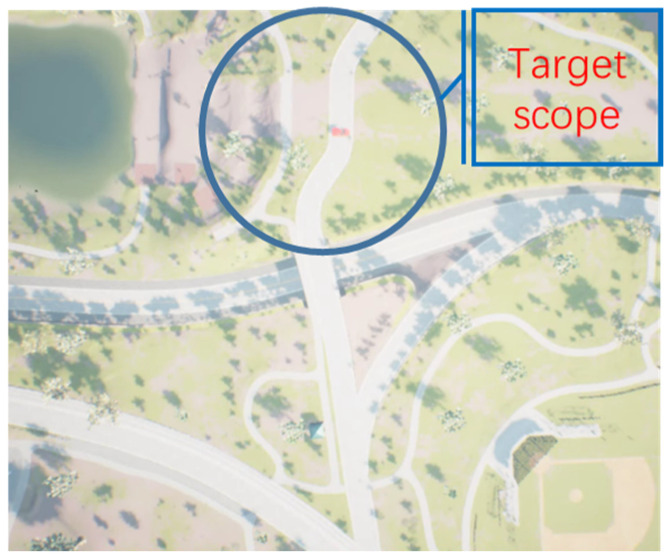
The reward function design method. Outside the target scope, the reward function is calculated by the distance between the target and the UAV. In the target scope, the UAV no longer knows the location of the target but uses a target recognition algorithm to detect the target, from which the reward is calculated.

Equation (8) shows the multi-UAV’s path planning reward. The position differential reward is used before the UAV flies to the edge of the target position range. The area differential reward of the recognition box is used when the UAV flies to the target range area. When the area of the recognition box is >0.01, the position between the UAV and the target is about 10 m, and this episode is set as a success. The mission will fail if any UAVs enter the defense area or collide with each other during the flight, as shown in Figure 10:

(9) $$reward=\left\{\begin{array}{ll} d^{t-1}-d^{t}, & d^{t}>20\\ \left(area^{t-1}-area^{t}\right)*200, & d^{t}<20\\ -20, & \mathrm{collision}\\ 20, & \mathrm{area}>0.01 \end{array}\right.$$

where $d_{t}$ represents the location of the UAV from the target at time t, and ${area}^{t}$ represents the area of the image recognition box at time t when the UAV flies to the target scope. When the UAVs collide with each other or enter the air defense zone, the reward is set to −20; when the area of the target identification box is >0.1, the reward is set to 20.

### 4.3. Hyper-Parameter

We implement our algorithm with Windows OS, PyTorch [33] deep learning framework, and Ray-distributed machine learning framework [34]. The input of the algorithm contains a forward view of the UAV, which we scaled to an 84 × 84 grayscale image to facilitate the computation of the neural network and the coordinate value and area value of the recognition box obtained after the YOLOv5 algorithm recognition.

We process the image using a three-layer convolutional neural network and concatenate it with the vector obtained by the YOLOv5 algorithm. Then, the concatenated vectors are then fitted using a fully connected neural network. Finally, the policy and Q values were obtained by the PPO algorithm. The hyper-parameters of the FF-PPO algorithm are given in Table 2.

### 4.4. Results, Validation, and Analysis

To test the feasibility of our algorithm, we conducted two sets of experiments.

(1) Unknowing the precise location of the target (Ours)

In this experiment, we assume that we do not know the exact location of the target and can only obtain the target location via object detection and then perform path planning.

(2) Knowing the precise location of the target (Precise Position)

In this experiment, PPO for reinforcement learning is also used, but we assume that the precise location of the target is already known, and there is no need to use the target detection algorithm to identify the target; moreover, we directly use the end-to-end method to realize the path planning operation.

**Figure 11:**
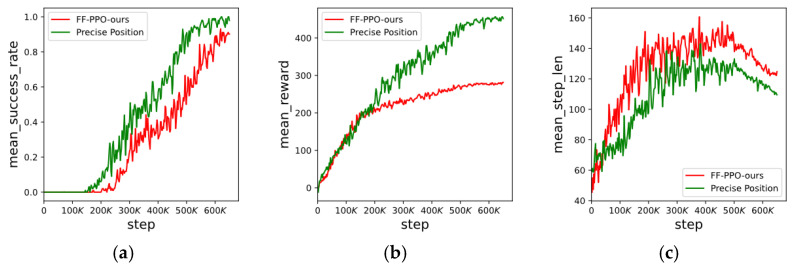
Training process. The horizontal axis is the number of training steps. (**a**) is the success rate curve, (**b**) is the reward curve, and (**c**) is the step length curve.

The training process is shown in Figure 11. Each time the three UAVs execute actions is called a step, and each success or failure of path planning is called an episode. The performance of the path planning algorithm gradually becomes superior as the number of experiments increases. We use the success rate of path planning, the average reward during the experiment, and the average number of steps required for each path planning as the index of the algorithm performance.

Figure 11a represents the process of reinforcement learning training. It is observed that the success rate is 0 at the beginning, indicating that the multi-UAV cannot complete the path planning task. As training proceeds, the success rate is close to 90%, indicating that path planning can be completed. Reinforcement learning is a dynamic programming method, so the planned path is not the same every time. The figure shows that the FF-PPO algorithm converges to 0.9 at 600 K, and the end-to-end method converges to 0.95 at 500 K. The success rate of the proposed method is slightly lower than the success rate of knowing the precise location of the target. The main reason is that the target recognition algorithm can encounter false recognition situations.

The curve of the reward function represents the convergence rate of the algorithm, as shown in Figure 11b. The UAVs obtain a high reward faster, indicating better algorithm performance. The rewards of the two methods finally reach convergence, but the rewards settings of the two methods are different because the area of the recognition box is used as the basis for the reward without the precise position of the target. In particular, when the UAV can fly close to the target position, the gap is more significant due to different reward design criteria.

Figure 11c shows the average number of steps per episode. Among them, the total step of the FF-PPO algorithm is slightly higher than that of the method with the exact position; the main reason is the false recognition of the image recognition algorithm. However, both algorithms converged in the end. At first, the UAVs could easily fly into the defense area, leading to failure. As the experiment proceeded, the UAVs gradually found the target position. Eventually, the UAVs learned to explore less and rationally fly to the target position; thus, the number of steps required for each scene gradually decreased. This means our proposed FF-PPO algorithm can solve the path planning problem of multiple UAVs at unknown target locations.

**Figure 12:**
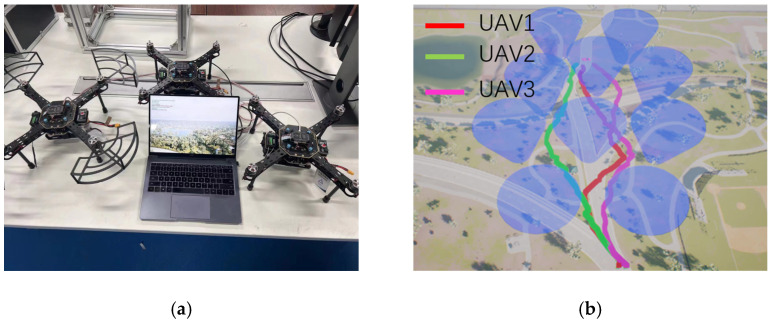
Algorithm verification. (**a**) shows the experimental configuration and (**b**) shows the FF-PPO algorithm’s path planning results. The red, green, and purple tracks represent the tracks of the three drones. It is worth emphasizing that the blue area is completely transparent to the UAVs, and the blue color in the figure is only for the convenience of the reader.

Figure 12a shows our procedure for validating the FF-PPO algorithm using a hardware-in-the-loop simulation. Figure 12b shows the trajectory of the cooperative path planning of the UAVs. The trajectories of the UAVs are not directly generated by the algorithm, but the reinforcement learning algorithm enables the UAVs in performing simple actions, completing the task of obstacle avoidance and flying to the target, and finally obtaining the result of path planning.

As observed in Figure 12b, at the beginning of the FF-PPO algorithm’s path planning stage, the UAVs separated from each other and passed through the gaps of different air defense zones to avoid collisions. When arriving near the target location, the UAVs determine the precise location of the target via the YOLOv5 algorithm. Finally, all UAVs bypassed the last air defense area and finally flew to the target position, completing the entire task of collaborative path planning.

## 5. Conclusions

This paper designs a UAV path planning algorithm with a visual navigation function to solve the problem of path planning in the case of an unknown target position in a GPS-denied environment. Due to the denial of GPS and communication, UAVs cannot obtain the precise position of the target. In the FF-PPO algorithm, the UAVs use the YOLOv5 algorithm for target recognition to obtain the position of the target and use the PPO algorithm for path planning. The experimental results show that the success rate of the autonomous navigation process converges to 90%, close to methods that use precision locations. The UAVs do not explore much but select a short, reasonable path. In addition, this paper used the PPO algorithm of an independent policy for the multi-UAV communication denial environment to achieve the distributed control of UAVs so that multiple UAVs can complete the collaborative path planning task. Finally, the hardware-in-the-loop experiment was carried out to verify the portability of the algorithm.

Since the independent policy cannot be applied to large clusters of UAVs, we will combine limited communication to achieve cooperative path planning with a larger number of UAVs in future studies.

## Figures and Tables

**Table 1:** Existing surveys related to the study of UAV path planning.

Publication	RL Algorithm	Year	Environment
Xie, R., et al. [8]	DQN	2020	3D-grid
Cui, Z.Y. [9]	DQN	2021	2D-grid
Liu, Y., et al. [10]	PPO	2020	3D-visual
Lowe, R., et al. [12]	MADDPG	2017	2D-grid
Xie, L., et al. [18]	DQN	2017	3D-visual
Kulkarni, T.D., et al. [20]	DQN	2016	3D-visual
Zhu, Y., et al. [21]	A3C	2017	3D-visual
Chen, L., et al. [22]	SAC	2017	3D-visual
Siriwardhana, S., et al. [23]	A3C	2019	3D-visual

**Table 2:** The hyper-parameters of the FF-PPO algorithm.

Parameters	Value
Gamma	0.995
Lambda	0.9
Learn rate	0.00025
SGD Minibatch Size	256
Train Batch Size	1024
Clip Param	0.3
Input size	84 × 84 × 1 + 5
Neural network structure	Conv1 [84,[4,4],4], Conv2 [42,[4,4],4], Conv3 [21,[5,5],2] Fcnet [512,256,64]

## Data Availability

Not applicable.
